# On the influence of relative humidity on the oxidation and hydrolysis of fresh and aged oil paints

**DOI:** 10.1038/s41598-019-41893-9

**Published:** 2019-04-02

**Authors:** Francesca Modugno, Fabiana Di Gianvincenzo, Ilaria Degano, Inez Dorothé van der Werf, Ilaria Bonaduce, Klaas Jan van den Berg

**Affiliations:** 1 Department of Chemistry and Industrial Chemistry, University of Pisa, Via Giuseppe Moruzzi 13, 56124 Pisa, IT Italy; 2 Department of Biology, University of Copenhagen, Øster Farimagsgade 5, 1353 Copenhagen, DK Denmark; 3 Cultural Heritage Agency of the Netherlands, Hobbemastraat 22, 1071 ZC Amsterdam, NL The Netherlands

## Abstract

Modern oil paintings are affected by conservation issues related to the oil paint formulations and to the fact that they are often unvarnished, and in direct contact with the environment. Understanding the evolution of the molecular composition of modern oil paint during ageing, under the influence of environmental factors, is fundamental for a better knowledge of degradation phenomena and risk factors affecting modern art. We investigated for the first time the influence of relative humidity on the chemical composition of modern oil paints during curing and artificial ageing. For this purpose, modern oil paint layers naturally aged for 10 years were further artificially aged in low and high relative humidity conditions. Moreover, the influence of RH% on the curing of fresh paint layers was studied. The paints used in the experiments are from three suppliers (Old Holland, Winsor&Newton, and Talens), and contain cadmium or cadmium zinc sulfide as main pigment. The changes in the composition of extracts of paint samples were investigated by direct electrospray mass spectrometry with a quadrupole-time of flight mass analyser (ESI-Q-ToF). The obtained mass spectral data were interpreted by means of principal component analysis (PCA) operated on a data set containing the relative abundance of ions associated to significant molecules present in the extracts, and also by calculating the ratios between the signals relative to fatty acids, dicarboxylic acids and acylglycerols, related to hydrolysis and oxidation phenomena. The same paint samples were also analysed, in bulk, by pyrolysis gas chromatography mass spectrometry (Py-GC/MS), achieving chemical information on the total lipid fraction. High performance liquid chromatography (HPLC) ESI-Q-ToF was carried out for the characterisation of the profile of free fatty acids (FFA) and acylglycerols, defining the nature of the oils used in the paint formulations, and for the determination of the degree of hydrolysis. This study demonstrated that relative humidity conditions significantly influence the chemical composition of the paints. Ageing under high RH% conditions produced an increase of the formation of dicarboxylic acids compared to ageing under low RH%, for all paints, in addition to a higher degree of hydrolysis, followed by evaporation of free fatty acids.

## Introduction

Drying oils have historically been used as binders in oil-based paints, thanks to their capability of forming a solid elastic film. The conservation of modern oil paintings, made in the twentieth- and twenty-first century, faces challenges that are different from oil paintings produced in the previous centuries. Many modern oil paintings are unvarnished^1^, meaning that the paint layer is unprotected from atmospheric impact, e.g. noxious gases^2^, light, moisture, and dirt. The absence of a varnish also entails that cleaning the surface of a modern oil painting is often a very delicate operation, having a direct effect on the paint surface. The presence of a vulnerable film on the paint surface (“skin of medium”) makes the paint particularly sensitive to unwanted changes^3^. Degradation phenomena such as efflorescence and the occurrence of water and solvent sensitivity are regularly observed^4–8^.

Unsaturated fatty acids, representing more than 80% of the total fatty acid composition of a drying oil, undergo oxidation and cross-linking reactions when exposed to light and oxygen, leading to the formation of a solid film, insoluble in water and in many organic solvents^9^. The curing mechanism, which leads to the formation of an oil paint network, is based on autoxidative radical chain reactions^10^. Polymerisation, given by the crosslinking reactions, and oxidation processes are in competition, and the latter may result in the oxidative cleavage of the fatty acids hydrocarbon chains and the formation of hydroxyacids, low molecular weight aldehydes and ketons, and α,ω-dicarboxylic acids with nonanedioic (azelaic acid) as the most abundant^10,11^. It has been demonstrated that certain metals present in pigments play a primary role in catalysing these reactions^12,13^ influencing the curing rate and mechanisms.

During ageing several other reactions may occur, the most important being the hydrolysis of the ester bonds of the triacylglycerols (TAGs)^14,15^. Cations in e.g. zinc, copper and lead containing pigments may react with free fatty acids and other carboxylic acid moieties present in the paint to form metal soaps agglomerates or ionomeric structures^9,16,17^. Some of these reactions may contribute to the formation of water sensitive oil paint surfaces. Research has led to hypotheses that link water sensitivity to the presence of carboxylate additives added during oil paints manufacturing because of their dispersive capabilities^15,18–20^. Recent research has shown that formation of water soluble salts is partly responsible for this phenomenon^2,6^. The formation of hydroxy groups and dicarboxylic acids can also play a role in the water sensitivity of modern oil paints, since these polar species are more hydrophilic than non-polar acylglycerols. Hydrolysis of the oil triacylglycerols, resulting in a lower degree of polymerization and coherence of the film, may be partially responsible for paint sensitivity to moisture observed in many modern oil paintings^17,21^. How these reactions, highly influenced by environmental conditions, contribute to make the paint film more sensitive to solvents is not yet completely understood^22^. Particularly, the influence of environmental moisture on the curing and degradation of paint has not been investigated in detail, but it is to be expected that also environmental parameters, including temperature, relative humidity, and composition of the atmosphere, can all contribute to the curing and ageing processes.

In this work, the influence of relative humidity on a range of aged and young paint films still in the process of curing was studied for the first time. This was done by monitoring the changes in composition of extracts of paint samples by direct electrospray mass spectrometry with a quadrupole-time of flight mass analyzer (ESI-Q-ToF). The same samples – without any prior extraction - were also analysed with pyrolysis gas chromatography mass spectrometry (Py-GC/MS) with tetramethylammonium hydroxide (TMAH). Preliminary investigation by high performance liquid chromatography (HPLC)-ESI-Q-ToF was also carried out for the characterisation of the profile of free fatty acids (FFA) and acylglycerols, determining the nature of the oils used in paint production.

Oil paint layers from three suppliers prepared in 2006 and naturally aged for 10 years were subjected to artificial ageing in different relative humidity (RH%) conditions. In addition, in 2016 the same paints were used to produce a new set of paint models, in order to study the influence of RH% on the early stages of the paints’ lifetime. Paints containing cadmium sulfide or cadmium zinc sulfide (cadmium yellow) as pigment have been selected for this study. The degradation of this pigment has been object of several literature studies^23–28^ whereas the degradation of the lipid binder has not been investigated in detail, to the best of our knowledge.

The study of the ageing processes was based on evaluation by means of Principal Components Analysis (PCA) of the abundances of selected ions in the ESI-Q-TOF mass spectra relative to ethanol extracts of the paint samples, in order to highlight alterations induced in the composition of the paint layers by artificial ageing. Moreover, ratios were calculated between the abundances of ions in the mass spectra of the extracts, associated to specific chemical species that are considered important chemical parameters to investigate the hydrolysis and oxidation degrees of oil paints.

Finally, analysis by pyrolysis coupled with gas chromatography and mass spectrometry (Py-GC/MS) was used to get a better insight into the nature of the oxidised species present in the paints.

The research was carried out within the framework of Cleaning Modern Oil Paints (CMOP), a collaborative European research project exploring challenges associated with the conservation of 20^th^ and 21^st^ century oil paintings (http://www.tate.org.uk/about/projects/cleaning-modern-oil-paints; http://www.jpi-culturalheritage.eu/joint-activities/joint-call/).

## Materials and Methods

### Paint materials

A set of oil paint reconstructions was cast in 2006 on Melinex sheet support using oil paint from three suppliers: Old Holland “classic oil colours”, Winsor&Newton “artists’ oil colours”, and Talens “Rembrandt oil colours” series^5,8^. The complete set has provided a useful collection of reference paint layers to study ageing and conservation issues of modern oils^14^. A selection, containing cadmium sulfide or cadmium zinc sulfide pigments, was included in this study and listed in Table 1.

**Table 1:** Oil paints layers investigated in this study. Pigment and extender composition taken from supplier information and^5^.

Trademark and paint type	Talens (TA) “Rembrandt Artists’ Quality”	Old Holland (OH) “Classic Oil Colour”	Winsor&Newton (WN) “Artists’ Oil Colour”
Pigment and extenders/additives composition	Cadmium Zinc Sulfide (PY35); aluminium stearate extender	Cadmium Sulfide; alumina pigment extender or coating; hydrogenated castor oil	Cadmium Zinc Sulfide (PY35), MgCO_3_, BaSO_4_; aluminium containing extender
Year of preparation and treatment:	2006: Artificial ageing at high (75%) and low (6%) RH%, in the dark at 70 °C, 12 weeks		
	2016: Curing at high (75%) and low (6%) RH%, ambient light and temperature, 9 weeks		

The investigated paint models were exposed to light-ageing in 2006 right after their preparation with a total artificial ageing time calculated to be equivalent to 24 years of exhibition in museum conditions (details are reported elsewhere)^5^, and subsequently stored indoor in daylight until 2012, when they were dismounted and kept in drawers and in the darkness. For this study two subsets of paints were subject to further artificial ageing at 70 °C, in the darkness, one at high (wet, W) and one at low (dry, D) relative humidity. In particular, the samples were placed in glass desiccators in an environment of 75% and 6% relative humidity, obtained using saturated NaCl and silica gel with anhydrous NaSO_4_, respectively. The artificial ageing treatment lasted 12 weeks. During this period samples for ESI-Q-ToF analyses were collected from the paint layers after 1, 2, 4, 6, 9 and 12 weeks of ageing time.

**Figure 1:**
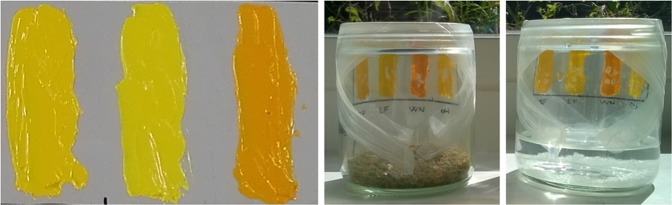
Paint layers and curing in dry and wet environment.

Another set of oil paint reconstructions (Table 1 and Fig. 1) was painted out in 2016 using the oil paints from the same paint tubes that were employed in 2006, in order to investigate the effects of environmental moisture on the curing process. The paint tubes were stored sealed and in the dark since 2006, and they were only opened for sampling procedures. The very first grams of paint were discarded in the preparation of the model samples of 2016.

These 2016 paint layers were divided in two sets and, a few days after making, left curing for 9 weeks in indoor ambient light and temperature, one at high and one at low RH%, by placing them in glass beakers covered with parafilm^©^ in an environment of 75% and 6% relative humidity, obtained as described before.

The thickness of the paint reconstructions prepared in 2006 was 90 μm (prepared using a metallic paint roller, which allows having uniform and known thickness). The thickness of the paint reconstructions prepared in 2016 was uniform, but not measured, as the reconstructions were prepared using a spatula. Sample collection (1 mm^2^, ~0.4 mg) was performed with a scalpel, taking care of collecting samples representative for the whole stratigraphy, from the bottom to the surface paint layer, in order to minimise the influence of the heterogeneity of curing processes between different sample collections.

### Reagents

For derivatization before HPLC-MS analysis, 2-hydrazinoquinoline (HQ), triphenylphosphine (TPP, purity 99%), and 2,2-dipyridyl disulfide (DPDS, purity 98%) were purchased from Sigma-Aldrich (USA). Solutions of 70.20 μg/g HQ, 69.00 μg/g DPDS and 79.50 μg/g TPP were prepared in acetonitrile (LC-MS grade, Sigma Aldrich) and stored at 4 °C in the dark. The solvents used as eluents were iso-propanol, water and methanol (HPLC-MS grade; Fluka). For calibration a stock fatty acid solution was prepared in acetone (HPLC grade; Sigma-Aldrich), and stored at 4 °C in the dark. It contained lauric acid (0.21 μg/g), suberic acid (0.22 μg/g), azelaic acid (0.16 μg/g), myristic acid (0.16 μg/g), sebacic acid (0.17 μg/g), palmitic acid (0.19 μg/g), oleic acid (0.26 μg/g), linoleic acid (0.20 μg/g), linolenic acid (0.18 μg/g) and stearic acid (0.26 μg/g). This solution was used to derive the calibration curves in the concentration range 3–400 ng/g. The acids were purchased from Sigma-Aldrich (purity > 99%). A second standard mixture solution was prepared in iso-propanol, containing the following commercial acylglycerols (all purity > 99%, Sigma-Aldrich, U.S.A.): monopalmitin (7.9 μg/g of mP), dipalmitin (7.0 μg/g of PP), trimyristin (8.0 μg/g of MMM), tripalmitin (10.0 μg/g of PPP), and tristearin (7.9 μg/g of SSS).

Ethanol Chromasolv for HPLC (≥99.3%) was used for extraction of samples before ESI-MS analysis.

Tetramethylammonium hydroxide (TMAH, Sigma-Aldrich) 5% in methanol (Chromasolv, LCMS grade) was used for *in-situ* thermally assisted hydrolysis and derivatisation in Py-GC/MS. All solvents were used without any further purification.

### Procedures and instrumentation

#### HPLC-ESI-Q-ToF

Fresh oil paints (directly from the paint tube, ~0.5 mg) and 2006 paint layers (~1 mg) were analysed by a recently developed analytical procedure^29^. The samples were added with 300 μL of a mixture of chloroform and hexane (CHCl_3_:HEX 3:2) in a Teflon vial. Microwave assisted-extraction (MLS-1200 MEGA Milestone (FKV, Sorisole (BG)) was performed for 25 minutes as follows: power 600 W, temperature 80 °C.

Sample aliquots of each extract were transferred in glass vials and dried under a stream of nitrogen. The derivatizing mixture was prepared by adding to the vial 20 μL of each of the solutions containing the derivatization reactants (20 μL 2,2-dipyridyl disulfide (DPDS) + 20 μL triphenylphosphine (TPP) + 20 μL 2-hydrazinoquinoline (HQ)), with a final volume of 60 μL. The reaction was performed in a 60 °C water bath for 6 hours.

Quantitative analyses of fatty acids against calibration curves in the range between 4 × 10^−3^ and 1 ppm were carried out using the solutions described in section 2.2, with a 1200 Infinity HPLC (Agilent Technologies, USA), coupled to a Jet Stream ESI interface (Agilent) and a Quadrupole-Time of Flight tandem mass spectrometer 6530 Infinity Q-ToF detector (Agilent Technologies). An Agilent Poroshell 120 EC-C18 column (3.0 mm × 50 mm, 2.7 µm) with a Zorbax eclipse plus C-18 guard column (4.6 mm × 12.5 mm, 5 µm) was used for the chromatographic separation. Acylglycerols were determined qualitatively in the same analytical run. The intra-day and inter-day precision RSD on the retention times and on peak areas was lower than 1% on both standard solutions and unknown samples, as described in detail in a paper dedicated to the optimization of the sample derivatization^29^.

The chromatographic conditions used were: injection volume 2 μL, column temperature 45 °C, flow rate 0.3 mL/min, drying gas (N_2_, purity > 98%) temperature 350 °C and flow 10 L/min, capillary voltage 4.5 kV, nebulizing gas pressure 35 psig, sheath gas (N_2_, purity > 98%) temperature 375 °C and flow 11 L/min. The eluents were: methanol/water 85:15 (eluent A) and 2-propanol (eluent B). The elution gradient was programmed as follows: 90% A for 5 min, followed by a linear gradient to 90% B in 25 min, then held for 5 min. Re-equilibration time for each analysis was 10 min. During the first part of the run (0–18 min), all ions were analysed in single mass spectrometry, with no fragmentation. Auto-MS-MS mode was employed in the second part of the run (18–40 min).

High resolution MS and MS/MS spectra were acquired in positive mode in the range 100–1700 m/z. The fragmentor was kept at 200 V, nozzle voltage 1000 V, skimmer 65 V, octapole RF 750 V, and the collision voltage for the Collision Induced Dissociation for MS/MS experiments was set at 50 V. The collision gas was nitrogen (purity 99.999%). The data were collected by auto MS/MS acquisition with a MS scan rate of 1.03 spectra/s and MS/MS scan rate of 1.05 spectra/s; only one precursor was acquired per cycle (relative threshold 0.010%), active exclusion after 3 spectra and 0.50 min (selection by abundance only). MassHunter Workstation Software (B.04.00) was used to control the HPLC and the mass spectrometer, for data acquisition, and for data analysis. Raw formulas corresponding to monoacylglycerols (MAGs), diacylglycerols (DAGs) and triacylglycerols (TAGs) were searched as [M + Na]^+^ ions by the “Find by Formula” algorithm provided by the software. For the derivatized FA, the raw formulas were searched as [M + H]^+^ ions by the “Find by Formula” algorithm. Formula matching was set at 20 ppm tolerance with a limit extraction range of 1.5 min and an area filter of 500 counts. Formula having an isotopic pattern score lower than 25% were discarded.

#### ESI-Q-ToF

An aliquot of the paint (~1 mm^2^, about 0.4 mg) was taken using a scalpel and put in a glass microinsert vial. Then 50 µL of ethanol were added. After mixing for 1 minute using a Vortex mixer (VWR International), the extraction was carried on for 1 hour at room temperature. Afterwards, the vial was centrifuged (Centrifuge VWR Microstar 17) for approximately 2 minutes, 13.500 rpm. An aliquot of 25 µL of the supernatant ethanol solution was thus transferred in a glass microinsert vial and 25 µL of 20 mM ammonium acetate (NH_4_Ac) in EtOH were added. The obtained solution was used for analysis.

Sampling and analysis were performed according to the following pattern: before starting the artificial ageing treatments (suffix in the sample labelling: 0w); every week for the first two weeks (suffix: 1w and 2w); every two weeks for the following four weeks (suffix: 4w and 6w); every three weeks for the following 6 weeks (suffix: 9w and 12w). The inter-day precision RSD on the peak intensities was lower than 10%^30^.

Analysis of the extracts was carried out using a Micromass Q-ToF 2 mass spectrometer equipped with a Micromass CapLC pump (Waters corporation, Milford, MA, U.S.A.), comprised of a ternary pump and autosampler. Samples were injected into the MS in a10 mM ammonium acetate (NH_4_Ac) ethanolic eluent, with a flow rate of 0.20 μL/min. The effluent of the CapLC was delivered to the ESI ion source with a picotip. All samples were measured in positive and negative ion modes. Operating conditions were: desolvation gas: nitrogen, 150 °C, 2 L/min; nebulizer gas: nitrogen, 1.5 L/min; cone gas: nitrogen, 2 L/min; collision gas: Argon. The ion source temperature was set at 80 °C, cone voltage 30 V, capillary voltage 3.0 kV, collision energy 10 V. The mass axis was calibrated using phosphoric acid. All MS data obtained were treated using MassLynx V4.0 (2004).

#### Py-GC/MS

The following paint samples were analysed with Py-GC/MS: the 12 weeks aged 2006 paints (high and low RH%; in the dark at 70 °C); the 9 weeks cured 2016 paints (high and low RH%; ambient light and temperature). These samples correspond to the final stage of the adopted artificial ageing and curing treatments.

A paint fragment (~1 mm^2^, about 0.4 mg) was taken using a scalpel and the sample material was added with a few drops of a 5% solution of tetramethylammonium hydroxide (TMAH) in methanol with tridecanoic acid internal standard and the suspension was transferred to a steel pyrolysis cup.

The pyrolysis unit used for ultrafast thermal desorption (UTD) was a Frontier Lab 3030D pyrolyser mounted on a Thermo Scientific Trace 1310 GC/ISQ mass spectrometer combination. UTD was performed by heating at 500 °C/min from 360 °C up to 700 °C. The analytical column was directly coupled to the pyrolyser via a home-made split device. A SLB-5ms (Supelco) column was used (length 20 m, int. diameter 0.18 mm, film thickness 0.18 μm). Helium was used as carrier with a constant flow of 0.9 mL/min and split ratio of 1:30. The temperature program was the following: 35 °C (1.5 min), heating at 60 °C/min to 100 °C, heating at 14 °C/min to 250 °C, heating at 6 °C/min to 315 °C (1.5 min). The column was directly coupled to the ion source of the mass spectrometer. The temperature of the interface was 240 °C, the temperature of the ion source was 220 °C. Mass spectra were recorded from 29 until 600 amu with a speed of 7 scans per second. Xcalibur 2.1, AMDIS 2.7 and MassLynx V4.0 softwares were used for collecting and processing of the data.

#### Principal component analysis

Principal components analysis (PCA) was performed using the software Xlstat2015 (Addinsoft, France) on the covariance matrix of the data.

## Results

### Characterisation of the oil binders by HPLC-MS

**Figure 2:**
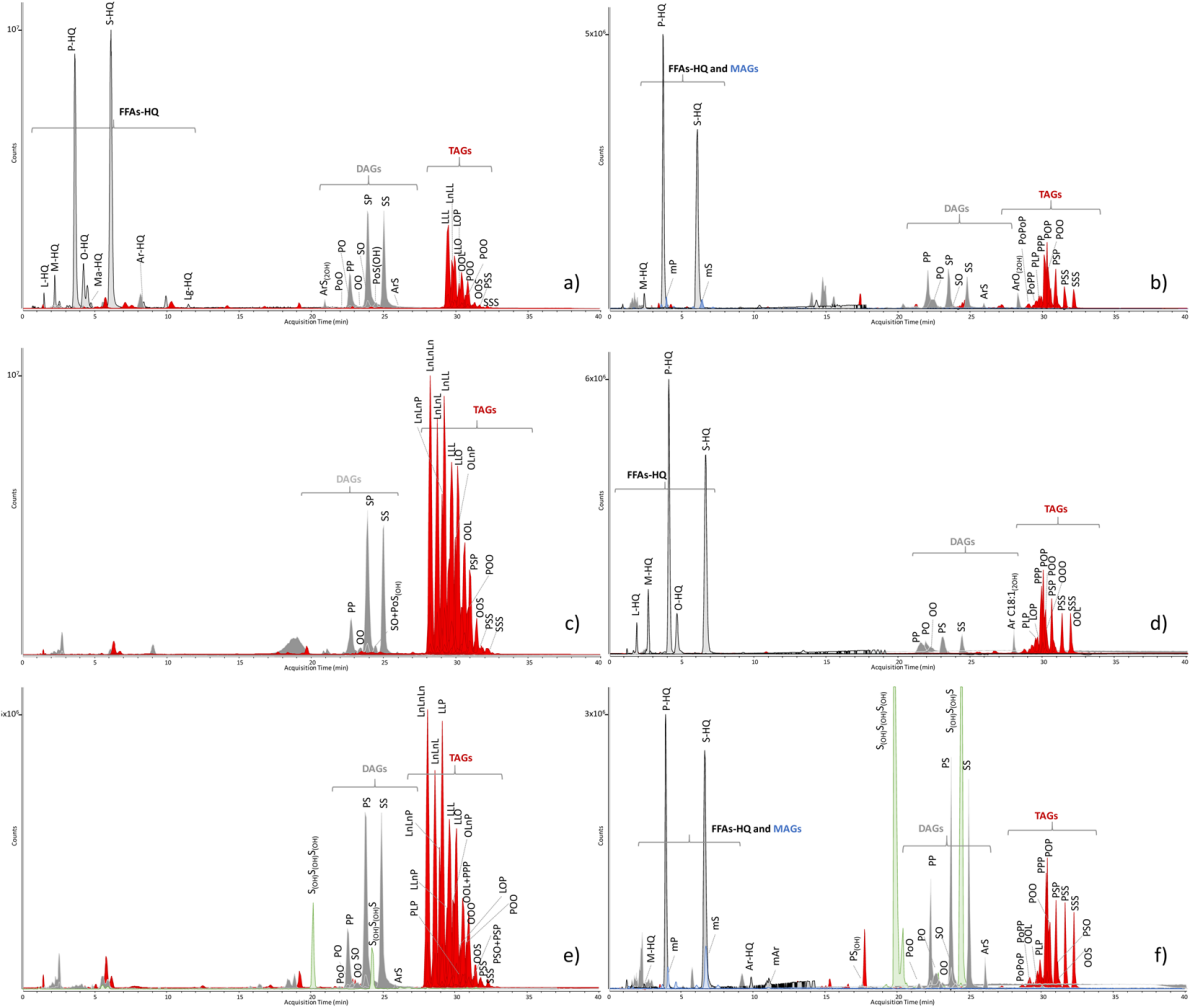
HPLC-ESI-Q-ToF extract ion chromatograms relative to fatty acids and acylglycerols in: (**a**) Talens fresh paint from the tube; (**b**) Talens 2006 paint layer; (**c**) Old Holland fresh paint from the tube; (**d**) Old Holland 2006 paint layer; (**e**) Winsor&Newton fresh paint from the tube; (**f**) Winsor&Newton 2006 paint layer. FAAs-HQ = HQ derivatives of free fatty acids (black line); MAGs = monoacylglycerols (blue line); DAGs = diacylglycerols (gray line); TAGs = triacylglycerols (red line); castor wax markers (green line). L = lauric acid; M = myristic acid; P = palmitic acid; Ma = margaric acid; O = oleic acid; L = lauric acid; S = stearic acid; Ar = arachidic acid; Lg = lignoceric acid. mP = monopalmitin; mS = monostearin. P: palmityl (C16:0); Po: palmitoleyl (C16:1); Ln: linolenyl (C18:3); L: linoleyl (C18:2); O: oleyl (C18:1); S: stearyl (C18:0); Ar: arachidyl (C20:0). (nOH) subscript indicates that the corresponding acyl chain contains n hydroxyl substituents.

A recently developed analytical method, based on reverse phase HPLC coupled with ESI-Q-ToF mass spectrometric detection^29^, was used for the determination of free fatty acids (FFA) and of the different classes of acylglycerols (TAGs, DAGs, and MAGs) in the 2006 paint samples before artificial ageing, and in the fresh paint directly from the tube (Fig. 2a–f).

Triacylglycerols were named according to the following fatty acid abbreviations: C_15_: pentadecanoyl (C_15:0_); P: palmityl (C_16:0_); Ln: linolenyl (C_18:3_); L: linoleyl (C_18:2_); O: oleyl (C_18:1_); S: stearyl (C_18:0_); Ar: arachidyl (C_20:0_); B: behenyl (C_22:0_); Li: lignoceryl (C_24:0_). The drying oils present in the different formulations were identified comparing the resulting triacylglycerols profiles with an in-house database and literature data^31^. Calibration curves relative to free fatty acids (FFAs) were used to determine the amount of FFA’s in each sample and are reported as ng of FFA per gram of sample (ppb) in Table 2.

**Table 2:** Ppb of FFAs determined by HPLC-ESI-Q-ToF analysis of Cd yellow fresh paints and 2006 paint layers Talens (TA), Old Holland (OH) and Winsor&Newton (WN).

	TA fresh	TA2006	OH fresh	OH 2006	WN fresh	WN 2006
Ppb of FFA	0.0	40.0	0.0	0.2	4.0	0.4

The data indicate that Talens and Old Holland paints contain linseed oil as binder, since the characteristic triacylglicerols LnLnLn, LnLnL, and LnLL were detected^32^. The Old Holland paint also contains castor wax^5^, identified by the presence of 2,3-di(12-hydroxy-octadecanoyloxy)propyl 12-hydroxy octadecanoate (tri,12-hydroxystearylglycerol, S_OH_S_OH_S_OH_) in the chromatogram.

The Winsor&Newton paint showed a different composition, featuring LLL, LLO, and POO that, together with the presence of triacylglycerols containing arachidic and lignoceric acids, allowed the identification of safflower oil.

As expected, linoleic and linolenic containing TAGs are absent in the extractable triacylglycerol fraction of the three 10-year-old 2006 paint layers, since polyunsaturated acyl chains are involved in autoxidative reactions. Oleic acid, which is less reactive than linoleic and linolenic acid^10^, is still present, as free fatty acid as well as within the acylglycerols.

The two linseed oil containing paints, Talens and Old Holland, featured negligible amounts of free fatty acids when analysed fresh from the tubes (Fig. 2a,c, Table 2), while free acids (in particular stearic and palmitic acid) were observed in the correspondent 10-years old paint layers (Fig. 2b,d), indicating that a certain degree of hydrolysis had taken place during natural ageing.

The safflower oil based Winsor&Newton paint contained a significant amount of free fatty acids in the fresh paint, higher than in the correspondent aged paint layer (Fig. 2e,f, and Table 2). This might be due to the fact that safflower oil was quite hydrolysed in the WN fresh paint, or that free fatty acids had been added to the paint formulation. The relatively lower content of FFA in the Winsor&Newton aged oil paint compared to the fresh paint can be ascribed to the fact that they have evaporated during 10 years of ageing^15^.

### PCA analysis of ESI-Q-ToF data

The 10 years’ aged paint layers were subject to artificial ageing in different RH% conditions in order to investigate the influence of environmental moisture on the ageing processes. After 10 years of natural ageing the main fraction of the oil is cross-linked. As a consequence, it has to be expected that no linoleic nor linolenic acid – the most reactive fatty acids in the oils^10^ – are still present in the oil. However, we cannot affirm that all double bonds in the linoleic and linolenic acids have been consumed, and thus we may assume that the cross-linked network still contains unsaturations. In order to promote the reactivity of the cured oil, and to make sure to be able to observe changes in the investigated time span, 70 °C were chosen as the operating artificial ageing temperature.

**Figure 3:**
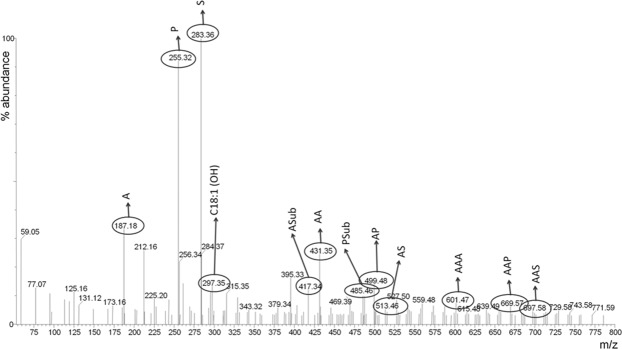
ESI-Q-TOF spectrum of 2006 Old Holland paint layer before artificial ageing. The main identified compounds are highlighted.

The extracts of the paint samples collected during artificial ageing were analysed by ESI-Q-ToF. Analyses were performed in both negative and positive mode, but only the results obtained in negative mode were informative and are thus reported in this work. The analyses were done on the paint extracts without a prior separation step, leading to a total superimposition mass spectrum of all ions. These ions are derived from the extractable fraction of the oil binder and of its oxidation and hydrolysis products. For the negative ion mode, the ions represent free fatty and dicarboxylic acids and mono-, di- and triacylglycerols containing dicarboxylic acids^21^. These molecules are in part different from those detected by HPLC-ESI-Q-ToF, due to the different operating MS conditions (positive and negative in the two cases) and to the different analytical procedures used prior to the MS analysis. The ESI-Q-ToF data refer only to the oil components that are soluble in ethanol, and thus non cross-linked, saponified or apolar. As an example, the mass spectrum of the extract of the Old Holland model paint cast in 2006 is reported in Fig. 3, highlighting the main identified compounds.

**Figure 4:**
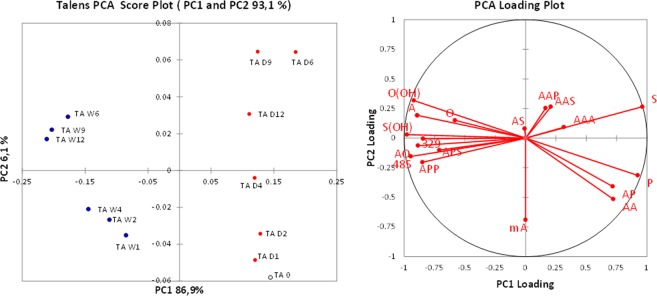
Score plot and loading plot obtained in the PCA of ESI-Q-ToF data for the 2006 Talens paint models during artificial ageing in wet (W, blue dots) and dry (D, red dots) conditions. The suffix number indicates the number of weeks of artificial ageing before sampling. TA 0 (empty dot) corresponds to the sample collected before starting the artificial ageing.

**Figure 5:**
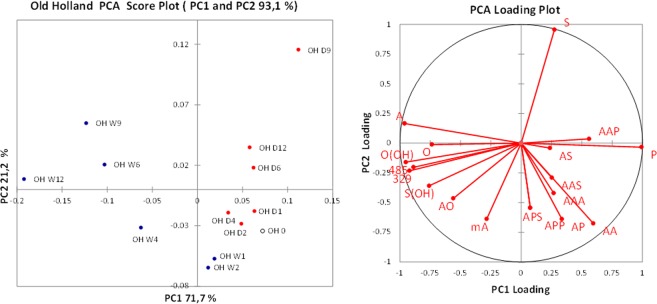
Score plot and loading plot obtained in the PCA of the ESI-Q-ToF data for the 2006 Old Holland paint models during artificial ageing in wet (W, blue dots) and dry (D, red dots) conditions. The suffix number indicates the number of weeks of artificial ageing before sampling. OH 0 (empty dot) corresponds to the sample collected before starting the artificial ageing.

**Figure 6:**
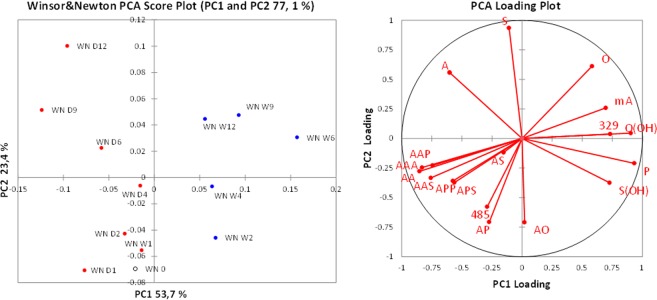
Score plot and loading plot obtained in the PCA of the ESI-Q-ToF data for the 2006 Winsor&Newton paint models during artificial ageing in wet (W, blue dots) and dry (D, red dots) conditions. The suffix number indicates the number of weeks of artificial ageing before sampling. WN 0 (empty dot) corresponds to the sample collected before starting the artificial ageing.

From the over 80 peaks at different *m/z* detected in the ESI-Q-ToF spectra, the intensities of the most important 17 ions were monitored during artificial ageing of the 2006 paint layers. In order to better highlight the differences between the samples, PCA was performed on the covariance matrix of the data. The relative intensities of the selected significant ions listed in Table 3 were used as input parameters. Row normalisation was applied to the data set before PCA, to remove the influence of the sample amount, since no quantitative analysis was performed by ESI-Q-ToF. The PCA results are reported in Figs 4–6 in the form of score plots and loading plots.

**Table 3:** Selected ions used for the PCA of the ESI-Q-ToF data. FA: fatty acid; MAG: monoacylglycerol; DAG: diacylglycerol; TAG: triacylglycerol. All ions are [M − H]^−^ except where indicated. *The DAGs corresponding to *m/z* 329 and 485 are still unidentified; their masses categorize them as diacylglycerols, but their exact structure has not been clarified. Nonetheless, they were included in the selection of ions because their trends appeared to be related to the ageing process.

m/z	Compound type	Compound	Abbreviation
187	FA	Azelaic acid (α,ω-nonandioic acid)	A
255	FA	Palmitic acid (hexadecanoic acid)	P
261	MAG	Azelaylglycerol	MA
281	FA	Oleic acid (9*Z*-octadecenoic acid)	O
283	FA	Stearic acid (octadecanoic acid)	S
297	FA	Hydroxy-oleic acid	O(OH)
299	FA	Hydroxy-stearic acid	S(OH)
329*	DAG	Unknown	329
431	DAG	Diazelayl glycerol ester	AA
485*	DAG	Unknown (polyhydroxylated)	485
499	DAG	Azelayl-, palmitylglycerol	AP
525	DAG	Azelayl-, oleylglycerol	AO
527	DAG	Azelayl-, stearylglycerol	AS
601	TAG	Triazelaylglycerol	AAA
669	TAG	Diazelayl-, palmitylglycerol	AAP
697	TAG	Diazelayl-, stearylglycerol	AAS
737	TAG	Azelayl, dipalmitylglycerol	APP
765	TAG	Azelayl-, palmityl-, stearylglycerol	APS

The difference in composition induced by ageing in different RH% conditions is the main source of variance in the data sets, and it is expressed by the first principal component for all the three data sets. The PCA score plots highlight a striking differentiation of the composition of the extracts between the paints artificially aged in a dry environment (D, red dots) and the paints artificially aged in a wet environment (W, blue dots), specifically differentiated along PC1.

Since each single measurement was undertaken at a different artificial ageing time, we expect the data to contain a significant amount of random error. Thus, we checked whether the effect of exposure to high RH on the value of PC1 was statistically significant. Moreover, the mean values of the PC1 for the paint layers aged in wet environment with the mean values of PC1 for the paint layers aged in dry environment were compared for the three paints. The t tests applied to the data confirmed that the average values of PC1 of paints aged in wet environment are statistically different from those of PC1 of paints aged in wet environment for all the three paints.

In the case of Talens (Fig. 4) and Old Holland (Fig. 5) paint layers, the samples from the dry environment score at positive values of PC1, together with the sample taken before treatment (week 0). In contrast, the samples from the wet environment tend to score at increasingly negative values of PC1 at longer ageing times.

To statistically confirm that the exposure to wet conditions caused a decrease in the PC1 value, at neat of random errors, the Pearson correlation index of PC1 values with the number of weeks of artificial ageing was calculated. For the paints artificially aged in wet environment, the Person correlation coefficient (R) between PC1 and week number was −0.77 for Talens and −0.98 for Old Holland, corresponding to a high and significant negative correlation. On the other side the R indices for the paint layers artificially aged in dry environment are −0.18 for Talens and 0.29 for Old Holland, showing that there is no correlation between PC1 and the weeks of exposure in dry environment.

The loading plots highlight that the differences induced by the wet environment are associated to a relatively higher content of hydroxylated and oxidised species in the extract: A, O(OH), S(OH) and the ions 329 and 485 have a significant negative loading on the PC1. Evaluation of the loading plots points out that the ageing at high RH% has favoured the formation of free hydroxylated species. Moreover, the fact that the loading of free azelaic acid (A) points in a different direction from the loadings of free palmitic and stearic acid (P and S) and of most of the azelaic-containing acylglycerols (AAP, AS, AAS, AAA, AA) shows that high RH% promotes the hydrolysis of the polar acylglycerols.

The data set obtained in the ESI-Q-ToF analysis of the extracts of the Winsor&Newton paint layers highlights a different behaviour upon artificial ageing. The Winsor&Newton paint already demonstrated to be different from the Talens and the Old Holland paints in the fact that it contains safflower oil instead of linseed oil, and that it shows a distinctively high degree of hydrolysis of the fresh paint (Table 2). The samples collected from the WN paint layer during artificial ageing in high and low RH% are differentiated on the first PC1 (Fig. 6), but in this case the samples from the dry environment (and the week 0 sample) score at negative values of PC1, while the samples from the wet environment score at positive values of PC1. The mean values of PC1 for the two groups of paint layers are statistically different; however, for Winsor&Newton paint the R correlation coefficients showed a moderate negative correlation between PC1 and number of weeks for dry ageing (R = −0.71), and only a moderate positive correlation for wet ageing (R = 0.54).

The difference in sign does not implicate a physical-chemical meaning, but the loading plot shows how in this case free azelaic acid is more abundant in the samples artificially aged in the dry environment, differently from what was observed for the other two suppliers. However, hydroxylated species and m/z 329 are correlated with ageing in a wet environment, as observed for Talens and Old Holland paints.

The variance explained by PC2 is much lower with respect to the PC1; however PC2 correlates very well with ageing time (Pearson correlation coefficients between PC2 and number of weeks of ageing: 0.82 and 0.86 for Talens aged in dry and wet conditions respectively; 0.77 and 0.78 for Old Holland aged in dry and wet conditions respectively; 0.99 and 0.94 for Winsor&Newton aged in dry and wet conditions respectively).

### Monitoring of characteristic ratios during artificial ageing

Given the fact that PCA of the ESI-Q-ToF data has highlighted a difference in composition between the paints that were artificially aged in different moisture conditions, the influence of environmental moisture on the ageing processes of the paint films was further investigated by monitoring the following set of characteristic ratios (evaluated as ratios between the relative abundances of the relevant ions reported in Table 3):

- A/P and A/S: azelaic to palmitic and azelaic to stearic acid ratios are indices of oil oxidation widely used in literature^11,33^;
- P/S: palmitic to stearic acid ratio was used as an indicator of the behaviour of saturated fatty acids^15^;
- A/AA: azelaic acid to glycerol diazelate ratio was studied as an indication of the degree of hydrolysis of the oil;
- FA_(OH)_/S: the ratio between the sum of the intensities of the signals assigned to hydroxy-octadecenoic (C18:1_(OH)_) and hydroxy-octadecanoic (C18:0_(OH)_) acids and the intensity of the signal of stearic acid was used as another index to study the oxidation degree of the oil.

**Figure 7:**
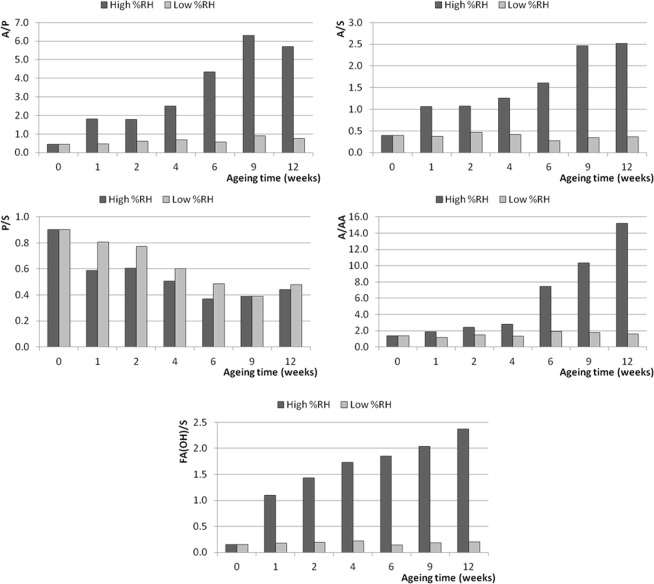
Trends of the characteristic ratios calculated from the ESI-Q-TOF spectra of the extracts of the 2006 Talens paint layers during artificial ageing at high and low RH%. 0 weeks corresponds to the samples before artificial ageing.

**Figure 8:**
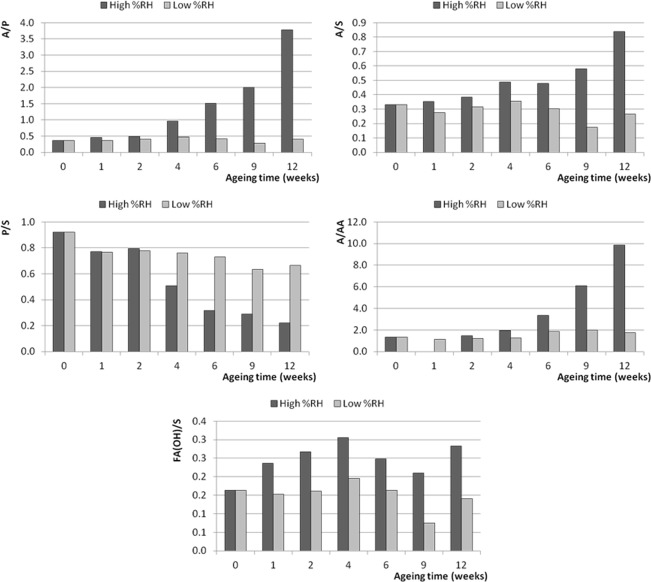
Trends of the characteristic ratios calculated from the ESI-Q-TOF spectra of the extracts of the 2006 Old Holland paint layers during artificial ageing at high and low RH%. 0 weeks corresponds to the samples before artificial ageing.

**Figure 9:**
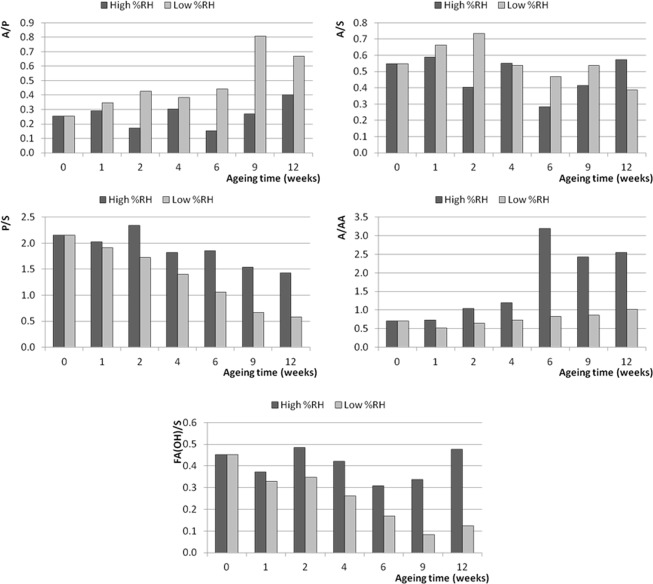
Trends of the characteristic ratios calculated from the ESI-Q-TOF spectra of the extracts of the 2006 Winsor&Newton paint layers during artificial ageing at high and low RH%. 0 weeks corresponds to the samples before artificial ageing.

The results are reported in Figs 7, 8 and 9 for the paints from the three suppliers - Talens, Old Holland and Winsor&Newton, respectively. In interpreting the results, it must be taken into account that different ionization yields of specific ions might affect the absolute value of the specific ratios calculated, and that these ESI-Q-TOF ratios refer to the ethanol extracts of the analysed paint samples and are therefore dependent on the extraction yields. It is, however, reasonable to assume that the ionisation and extraction yields did not vary significantly between one analysis and the other, and within the same sample subject to increasing ageing. The evaluation of the changes of the values of the calculated ratios during artificial ageing can thus be related to chemical changes occurring in the paint layers under the influence of the wet and the dry environment at high temperature (70 °C).

The variations of the observed parameters during artificial ageing confirm the preview achieved by PCA: the level of environmental moisture dramatically influences the chemical composition of the paint, as exemplified by the values determined for the Talens paint layer (Fig. 7). In particular, during artificial ageing under dry conditions the calculated ratios remain fairly stable during the 12 weeks. The only significant trend observed at low RH% is actually a decrease of the P/S ratios (from 0.9 to 0.5 in 12 weeks for the Talens paint, from 0.9 to 0.6 for the Old Holland paint, and from 2.1 to 0.6 for the Winsor&Newton paint). A comparable decrease of P/S is observed in wet environment (with final values of 0.4 for the Talens paint, 0.2 for the Old Holland paint, and 1.4 for the Winsor&Newton paint after 12 weeks). The decrease in P/S can be ascribed to the evaporation of free fatty acids at elevated temperatures (artificial ageing was performed at 70 °C), which affects palmitic acid more than stearic acid^34,35^.

The values of the other calculated ratios evolved very differently in dependence of environmental moisture. A clear increase of the A/P and A/S ratios with ageing in high RH% conditions was observed for the Talens and Old Holland paints, whereas for the Winsor&Newton no clear trend can be found. The increase in free azelaic acid can be ascribed to preferential hydrolysis of more polar acylglycerols containing dicarboxylic moieties, but it can also be due to oxidation of the remaining oleic acid and other unsaturated fatty acid moieties taking place at high RH%, as confirmed by the increase of the FA(OH)/S ratios. The A/AA ratio also clearly increased during ageing in high RH% conditions, indicating a higher degree of hydrolysis as ageing proceeds in humid environment.

As mentioned, the described increase in the A/P, A/S, A/AA and FA(OH)/S ratios in humid environment was substantial for the two linseed oil-based paints Talens (Fig. 7) and Old Holland (Fig. 8). The trend was less evident for the safflower oil-based Winsor&Newton paint (Fig. 9), where the A/P ratio increased more in the low RH% than in wet RH%.

Comparison of the paints produced by the different suppliers suggests that linseed oil-containing paints are more prone to hydrolysis promoted by wet environment than the safflower oil-containing paint (Winsor&Newton), which on the other side underwent oxidation with formation of azelaic acid during artificial ageing in both wet and dry environment. Preferential evaporation of palmitic acid also slightly contributes to the increase of A/P ratio.

It is important to underline that the differences in behaviour observed among the paint from the different suppliers can be ascribed not only to the different composition of the lipid binders highlighted by HPLC-MS analysis, but can also partly derive from the influence of other components of the formulation, as additives, or a different composition of the CdS pigment. The influence of paint additives on ageing and curing of modern oil paint has been studied in the course of the CMOP project^14,36^ and is beyond the scope of the present investigation.

### Effect of RH% on the curing of young paints

**Figure 10:**
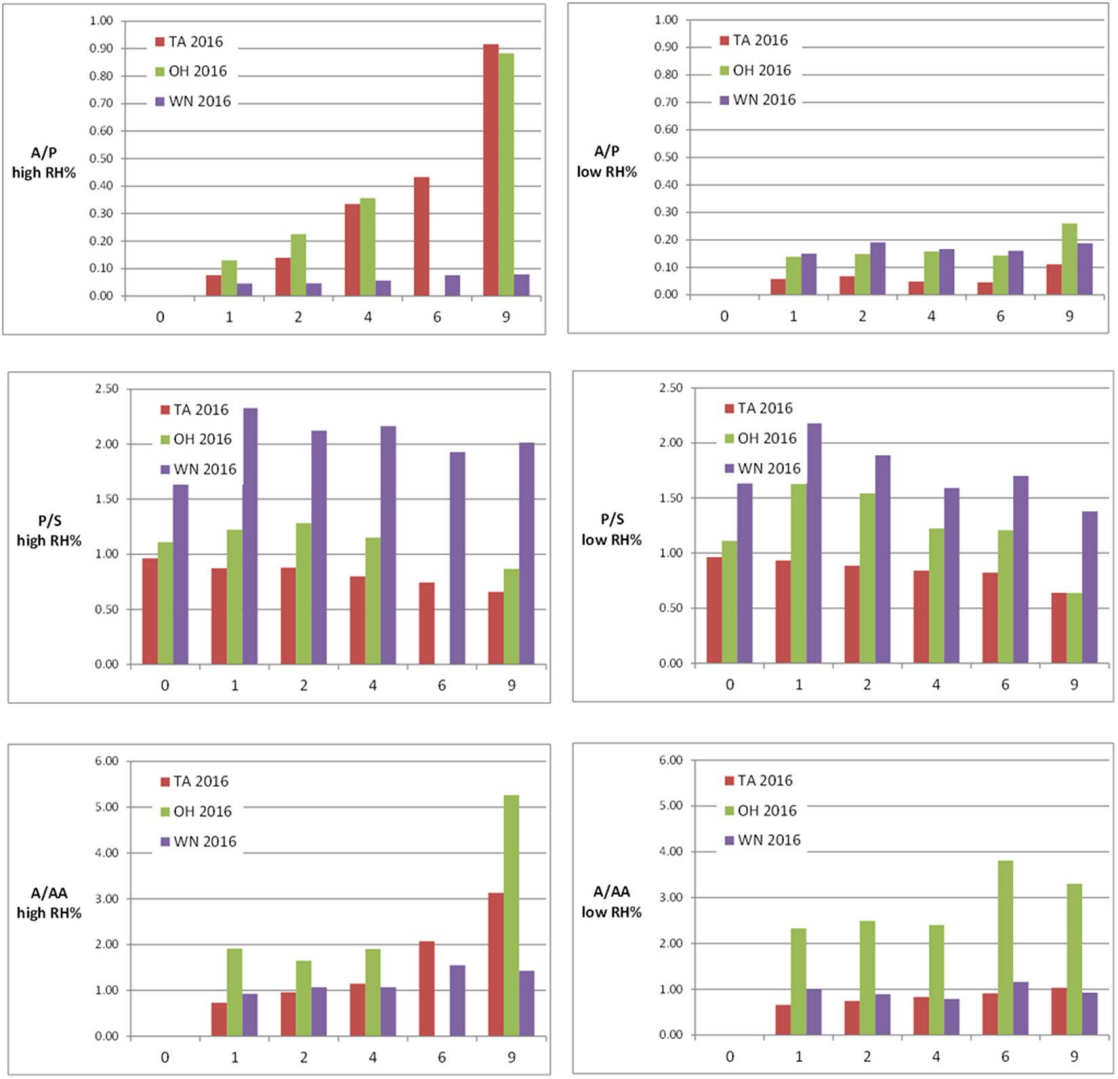
A/P, P/S and A/AA ratios of the extracts of the Talens (TA), Old Holland (OH) and Winsor&Newton (WN) paints prepared in 2016 and cured at room temperature, in different RH% conditions for 9 weeks (the data corresponding to the OH paint cured 6 weeks in high RH% environment could not be acquired).

Paint layers produced in 2016 with the same paints used to prepare the paint layers in 2006 were monitored by ESI-Q-ToF during curing for 9 weeks in different RH% environments at room temperature and the evolution of the values of the A/P, P/S and A/AA ratios are reported in Fig. 10.

No azelaic acid was detected by ESI-Q-ToF in the extracts of the 2016 paints at the beginning of curing (week 0). After 1 week of curing - in both RH% conditions – formation of azelaic acid is observed in all the paints, with a remarkable increasing trend during the 9 weeks, in particular for the linseed oil based paints (TA and OH) in wet environment, reaching a final value of A/P = 0.9. In the same paints cured in dry environment, the final value of A/P is substantially lower (0.1 for TA and 0.3 for OH). This result indicates the occurrence of a phenomenon never reported before in the literature: the oxidative processes leading to the formation of dicarboxylic acids during curing proceed at significantly higher speed in high relative humidity conditions than in dry conditions.

Food research has evidenced that water may play a fundamental role in determining the pathway of autoxidation of lipids^37,38^: in very dry environments oxidation rates are very high, at low levels of water activity (monolayer value) the oxidation rate reaches a minimum, and at relatively high levels of water activity high oxidation rates are observed. When metal salts are present, water may favour the dissociation and exert a prooxidative effect^39^. On the other hand, the role of water in the oxidation processes of lipids may also be related to association colloids, such as reverse micelles, that were shown to be formed in edible oils during the initiation phase, as amphiphilic molecules and hydroperoxides begin to aggregate. New evidences have shown that in bulk oils the crucial site of oxidation is not the air-oil interface, but the water-lipid phase of association colloids that are generated with traces of water and surface active molecules (free acids, mono- and diacylglycerols, hydroperoxides, etc.)^40^. Water content and mobility could influence the size and number of association colloids as well as the activity of reactants at the water-lipid interface or in the water core. However, more studies are needed to improve our understanding of the supra-molecular chemistry of lipid oxidation, and further research is also needed to investigate if the faster oxidation and cleavage of double bonds leading to a higher yield of dicarboxylic acids in short time is accompanied by a different degree of reticulation and cross-linking.

In the Winsor&Newton paint, containing semi-siccative safflower oil, the formation of free azelaic acid during the first 9 weeks of curing is much more limited than in the linseed oil based paints, with final values of A/P of 0.1 for high RH% and 0.2 for low RH%. Since linseed oil contains more polyunsaturated fatty acids than safflower oil, linseed oil-containing paints are expected to show a higher degree of oxidation than safflower oil-containing paint. However, it has to be underlined that in the case of the Winsor&Newton paint the low A/P values are influenced also by the non-negligible amount of free palmitic acid originally present in the paint (see Table 2).

The P/S ratio showed moderate oscillations during curing, with a certain tendency to decrease ascribable to preferential evaporation of palmitic acid, which revealed to be more pronounced in low RH% environment, and in particular for the Winsor&Newton paint (rich in free acids).

The A/AA ratio does not show a clear trend during curing at low RH%, whereas a certain increase was observed after 6 and 9 weeks of high RH% curing, highlighting once again the influence of environmental moisture in promoting hydrolysis, not only during ageing but also in the first stages of curing.

### Py-GC/MS

In order not to limit the investigation to the composition of the extractable fractions carried out by ESI-Q-ToF, Py-GC/MS analysis with TMAH (transesterification/methylation) of solid microsamples of bulk paint was performed at the final stages of the artificial ageing and curing treatments. This approach enables gathering information on both the extractable and non-extractable/reticulated fractions, containing free fatty acids, as well as unpolymerised fatty and dicarboxylic acids bound as acylglycerols or present as metal carboxylates^41^. The A/P, A/S,P/S and FA_(OH)_/S ratios, based on the total ion chromatogram (TIC) and extracted ion chromatogram (EIC) peak areas, were calculated and are reported in Table 4. As a measure of the degree of oxidation, the A/P ratios derived from Py-GC/MS data are to be considered more representative of the overall degree of oxidation of the paint film than the same ratios derived from the ESI-Q-TOF data set (which only relates to the polar extractable organic components of the paint). The A/AA ratio could not be determined with Py-GC/MS due to the hydrolysis of glyceroldiazelate into azelaic acid and glycerol by TMAH.

**Table 4:** Characteristic ratios calculated from the Py-GC/MS TIC and EIC chromatograms of the 2016 paint layers after 9 weeks of curing and of the 2006 paint layers after 12 weeks of artificial ageing, at high and low RH%. Talens (TA), Old Holland (OH) and Winsor&Newton (WN). Replicate measurements performed on one sample indicated typical RSD of 3%.

	TA 2016		TA 2006		OH 2016		OH 2006		WN 2016		WN 2006	
	High RH%	Low RH%	High RH%	Low RH%	High RH%	Low RH%	High RH%	Low RH%	High RH%	Low RH%	High RH%	Low RH%
A/P	0.6	0.7	2.2	1.0	1.7	1.7	1.5	0.7	1.1	1.4	1.0	1.3
A/S	0.6	0.7	1.0	0.9	2.0	2.0	1.0	0.7	2.1	2.7	1.9	2.4
P/S	1.0	1.0	0.4	0.9	1.2	1.2	0.7	0.9	2.0	2.0	1.8	1.8
FA_(OH)_/S	0.5	0.2	0.3	0.2	0.7	0.6	0.6	0.2	1.1	1.2	0.7	0.6

Concerning the oxidation products of octadecenoic and octadecanoic acid (FA_(OH)_), the following compounds were identified (as methyl ethers/esters): 8-hydroxyoctadec-9-enoic acid, 11-hydroxyoctadec-9-enoic acid, 9-hydroxyoctadec-10-enoic acid/ 10-hydroxyoctadec-8-enoic acid, 10-hydroxyoctadecanoic acid, 9,10-epoxyoctadecanoic acid, and 9,10-dihydroxyoctadecanoic acid. Among these, the most abundant were 9-hydroxyoctadec-10-enoic acid/ 10-hydroxyoctadec-8-enoic acid and 9,10-dihydroxyoctadecanoic acid. The epoxy compound peak resulted generally much lower.

**Figure 11:**
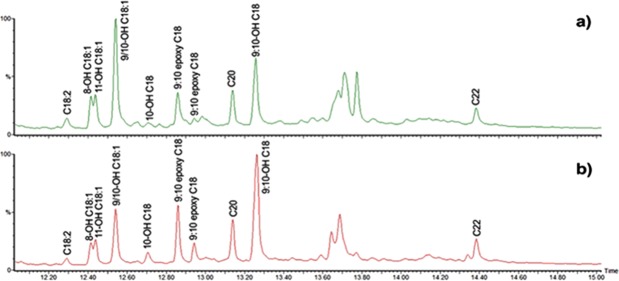
Py-GC/MS total ion chromatograms of Winsor&Newton paint reconstructions (2016) cured at (**a**) high and (**b**) low RH%. n-OH Cx = n hydroxy linear saturated monocarboxylic acid with x carbon atoms. n-OH Cx:y = n-hydroxy linear monocarboxylic acid with x carbon atoms and y unsaturations. The peaks labelled a-d correspond to unidentified oxidised fatty acids.

The Py-GC/MS analyses of the young paints evidenced the presence of some unidentified oxidised fatty acids, eluting between 13.6 and 13.8 minutes (Fig. 11), which were found to be more abundant at high RH% as compared with low RH% conditions, at the expense of 9,10-epoxyoctadecanoic acid and 9,10-dihydroxyoctadecanoic acid. It should also be evidenced that their relative abundance seems to vary as well, indicating that the level of environmental humidity influences the formation of different oxidation products.

Moreover, the pyrograms showed the presence of peaks ascribable to doubly unsaturated fatty acids. As no linoleic acid was detected in any of the ESI-MS nor HPLC-MS chromatograms, we can conclude that these derive from the transesterification of unsaturated fatty acids covalently bound to the polymeric network, confirming that double bonds are still present in the paint film, and may thus be subject to further reactions.

Py-GC/MS data strongly support the findings of the ESI-MS investigations previously described for the artificially aged Talens and Old Holland paints. Ageing at high RH% resulted in a decrease of P/S, an increase of A/P and A/S, and a significantly higher amount of oxidised octadecenoic and octadecanoic acids with respect to the paints aged in dry conditions. For example, the pyrolytic A/P ratio of the TA paint was 2.2 (Table 4) after artificial ageing in wet conditions, and 1.0 in dry conditions; the pyrolytic P/S ratios were ∼0.9 at low RH% and ∼0.4 at high RH%. The influence of RH% on the P/S, A/P and A/S ratios of the cured 2016 paints is less evident.

The WN paints – both the cured 2016 paints and the aged 2006 paints – are only slightly affected by the presence of high RH%. Artificial ageing in a dry environment caused a more marked increase of the A/P and A/S ratios than artificial ageing in a humid environment, whereas no significant difference was observed between the P/S ratios at low and high RH%. The FA_(OH)_/S ratio is slightly higher at high RH%. These differences must be ascribed, on one hand, to the higher degree of hydrolysis of safflower oil in the paint tube, and on the other hand to the fact that safflower oil contains only oleic and linoleic acid, while in linseed oil also significant amounts of the most reactive linolenic acid are present^10^.

## Conclusions

This study was aimed at understanding the effect of relative humidity on curing and ageing of oil paintings. In particular, the study was focussed on modern oil paintings, which are proving unprecedented and challenging conservation problems, in order to provide fundamental knowledge to be used to prevent and slow-down the decay. Three commercial oil paints containing cadmium sulfide (cadmium yellow) were used to prepare model paints: a first set in 2006, and another set in 2016. These were exposed to different conditions of relative humidity (dry and humid environments). The model paints from 2006 were artificially aged at 70 °C, in order to accelerate ageing, while the model paints from 2016 were left to cure at ambient temperature. Their chemical composition was investigated by HPLC-ESI-Q-ToF and Py-GC/MS. The paint layers from 2006 still contain a portion of double bonds, present both as unreacted oleic acid (as free fatty acid and as acylglycerol), and as unsaturated fatty acids covalently bound to the polymeric network. These unsaturations may undergo chemical reactions, including autoxidation. The presence of characteristic polyunsaturated triacylglycerols enabled us to distinguish paints containing linseed oil (Talens and Old Holland), characterized by high concentrations of linolenic acid and linoleic acid, and paints containing safflower oil (Winsor&Newton), containing high amounts of linoleic acid and other specific triacylglycerols. In addition, HPLC-ESI-Q-TOF highlighted the presence of free fatty acids in the Winsor&Newton paint and of castor wax in the Old Holland paint. The different chemical composition of the oils reflects in their siccative properties, as linseed oil is a drying oil and safflower oil is a semi-drying oil.

The changes taking place upon curing and artificial ageing were monitored by ESI-Q-ToF and data were elaborated with the support of principal component analysis. Results clearly indicate that high levels of relative humidity catalyse acylglycerol hydrolysis, and lead to the formation of relatively higher amounts of oxidation products, including hydroxylated fatty acids and dicarboxylic acids. The type of oil proved to be responsible for a different response of the model paints to high levels of relative humidity: upon curing the safflower oil-containing paint is less susceptible to high relative humidity, and lower amounts of dicarboxylic acids and hydroxylated species are formed.

Although food research had previously highlighted that water plays a role in the autoxidation processes of lipids, to the best of our knowledge, the results presented here are the first to prove that relative humidity is an important factor in determining reaction pathways during ageing and curing of oil paints. In addition, the influence on the oxidative chain scission of the unsaturated fatty acids have not been shown before. The obtained data point to an oxidative effect of high levels of relative humidity, possibly via mobilisation of radicals and activation of pigment catalytic activity. However, further experiments are necessary to understand the mechanisms of action, as studying the effects of intermediate levels of humidity, and of variations of RH% levels and of temperature. Moreover, potentially relevant parameters as the presence and amount of additives in the formulation, not to mention paint thickness, are worth to be investigated in future studies.

The study highlights the potential importance of the findings presented in this paper for conservation of painted works of art, most notably for oil paintings as well as for chemically related alkyd paints^3^. The demonstrated influence of a high relative humidity during curing and ageing of oil paints can be expected to lead to paints that are less cross-linked and may in turn be more susceptible to solvent sensitivity^3,6,21,22^.
